# Association Between Sarcopenic Obesity and Activities of Daily Living in Individuals with Spinal Cord Injury

**DOI:** 10.3390/jcm13237071

**Published:** 2024-11-22

**Authors:** Ryu Ishimoto, Hirotaka Mutsuzaki, Yukiyo Shimizu, Ryoko Takeuchi, Shuji Matsumoto, Yasushi Hada

**Affiliations:** 1 Graduate School of Comprehensive Human Sciences, University of Tsukuba, Tsukuba 305-8575, Japan; ishimotori@ipu.ac.jp; 2 Department of Rehabilitation Medicine, Ibaraki Prefectural University of Health Sciences Hospital, Ami 300-0331, Japan; 3 Center for Medical Science, Ibaraki Prefectural University of Health Sciences, Ami 300-0394, Japan; 4 Department of Orthopaedic Surgery, Ibaraki Prefectural University of Health Sciences Hospital, Ami 300-0331, Japan; 5 Department of Rehabilitation Medicine, Institute of Medicine, University of Tsukuba, Tsukuba 305-8575, Japan

**Keywords:** spinal cord injury, sarcopenic obesity, activities of daily living, functional independence measure, dual-energy X-ray absorptiometry

## Abstract

**Background/Objectives**: Sarcopenic obesity adversely affects physical function and activities of daily living (ADL) in older individuals and patients undergoing rehabilitation. This condition is also common in individuals with spinal cord injury (SCI); however, its relationship with ADL in this group remains unclear. Hence, this study examined the association between sarcopenic obesity and ADL in individuals with SCI. **Methods**: This retrospective cross-sectional study identified sarcopenia using the low skeletal muscle mass index (SMI) and Asian Working Group for Sarcopenia reference values. Obesity was defined as a body fat percentage (%BF) exceeding 25% in men and 35% in women. Sarcopenic obesity was identified when both the sarcopenia and obesity criteria were met. The primary outcome, ADL, was measured using the Functional Independence Measure (FIM). Multiple linear regression models were used to analyze the associations among the SMI, %BF, and FIM scores, after adjusting for age, sex, lesion level, injury severity, comorbidities, and injury duration. **Results**: Of 82 participants (median age: 63.5 years; 18.3% women), 62.2% had sarcopenic obesity. Participants with sarcopenic obesity (54 vs. 69 points, *p* = 0.006) had significantly lower FIM motor scores than those without this condition. Multiple linear regression analysis revealed that SMI (β = 0.416, *p* < 0.001) and %BF (β = −0.325, *p* = 0.009) were independently associated with the FIM motor scores. **Conclusions**: Decreased SMI and increased %BF in patients with SCI were independently associated with decreased ADL independence. Routine body composition assessments are necessary for early detection and intervention in this population.

## 1. Introduction

Sarcopenia is characterized by an age-related decline in skeletal muscle mass, accompanied by a loss of muscle strength or physical function [1]. When sarcopenia occurs alongside obesity, it is termed sarcopenic obesity. The prevalence of sarcopenic obesity varies, depending on the criteria, individual characteristics, and demographics, ranging from 1.3% to 20.3% in male individuals and 0.8% to 33.5% in female individuals among community-dwelling older individuals [2,3]. Among patients recovering from stroke, musculoskeletal disease, and hospital-associated deconditioning in rehabilitation settings, the prevalence of sarcopenic obesity is between 20% and 30% [4,5].

Sarcopenic obesity is prevalent in individuals with spinal cord injury (SCI). Secondary to the neurological changes after SCI, muscle atrophy of the paralyzed region below the neurological level of injury begins within weeks of SCI onset [6,7,8,9]. Muscle atrophy is concomitant with an increase in intramuscular fat accumulation that persists for several months [7]. Furthermore, body composition undergoes additional changes during the subacute to chronic phases. Patients are at high risk of inactivity and undernutrition due to surgery, prolonged bed rest, and emerging complications, which likely accelerate muscle atrophy due to deconditioning. Muscle atrophy leads to a decline in basal metabolism, and the resting basal metabolic rate in patients with SCI is reported to be 14–27% lower than that in healthy individuals [10,11,12]. Disability restricts the activities of daily living (ADL), which likely accelerates the decline in skeletal muscle mass and total daily energy expenditure. A positive energy balance occurs without adjustments in energy consumption, which likely increases fat tissue mass. Therefore, individuals with SCI are susceptible to developing sarcopenic obesity. The prevalence of sarcopenic obesity in individuals with SCI is relatively high, ranging from 41.9% to 63.9% [13,14].

The high prevalence of sarcopenic obesity in individuals with SCI is noteworthy because these conditions may be associated with multiple health problems, including diabetes, hyperlipidemia, metabolic syndromes, and cardiovascular diseases [10,13,15,16]. Moreover, recent studies have reported that sarcopenic obesity may negatively affect physical function and ADL in community-dwelling older individuals, as well as in patients in rehabilitation settings [4,5,17,18]. Yoshimura et al. investigated the effect of sarcopenic obesity on ADL in 971 Japanese patients recovering from stroke, musculoskeletal disease, and hospital-associated deconditioning in a convalescent rehabilitation ward [4]. They reported a negative association between sarcopenic obesity and functional improvement. Similarly, Matsushita et al. investigated the links between sarcopenic obesity and ADL in older patients (aged ≥ 65 years) recovering from stroke in a convalescent rehabilitation ward and revealed an independent association between sarcopenic obesity and lower ADL capabilities [5]. Despite the growing interest in this topic, to the best of our knowledge, no studies have investigated the association between sarcopenic obesity and ADL in individuals with SCI, leaving this relationship unexplored.

Clarifying the relationship between sarcopenic obesity and ADL in individuals with SCI may provide insights that could improve the care and therapeutic interventions for those with SCI. Hence, we aimed to investigate the associations between sarcopenic obesity and ADL in individuals with chronic SCI. Based on the results of previous studies, we hypothesized that sarcopenic obesity negatively affects ADL [4,5].

## 2. Materials and Methods

### 2.1. Study Design, Participants, and Setting

This retrospective cross-sectional study was performed at the Ibaraki Prefectural University of Health Sciences Hospital, a rehabilitation hospital with a 120-bed capacity.

Participants eligible for inclusion in this study were individuals aged ≥ 20 years (legal adults) with chronic SCI (lasting ≥ 6 months) and stable ADL ability, in stable medical condition, admitted to our hospital for post-acute rehabilitation, routine physical assessments, or fitness enhancement, and who underwent whole-body dual-energy X-ray absorptiometry (DXA) for body composition assessment between September 2016 and May 2024. The exclusion criteria were age < 20 years (legal minors), dysphagia, and missing data.

**Figure 1:**
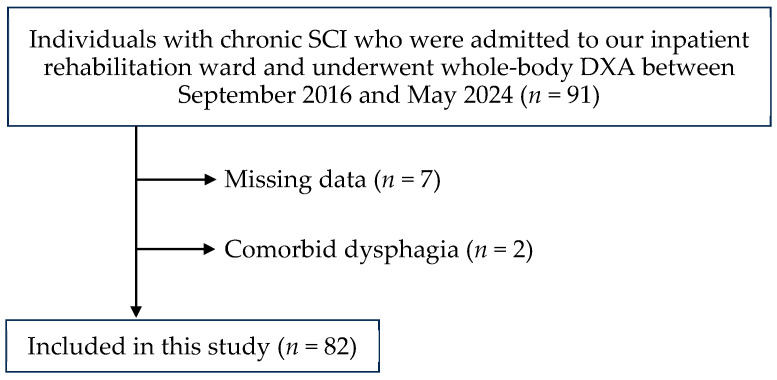
Recruitment flowchart of participants.

Figure 1 depicts the recruitment flowchart for the participants. During the study period, 91 individuals with chronic SCI underwent body composition assessments using DXA. Seven patients with missing data and two patients with dysphagia were excluded from the analysis. The remaining 82 patients were eligible for inclusion in this study.

### 2.2. Data Collection

Basic data, such as age, sex, diagnosis, and duration of injury (in days) were collected from medical chart reviews. The American Spinal Injury Association Impairment Scale (AIS) criteria, based on the International Standards for Neurological Classification of SCI, were used to assess lesion levels (tetraplegia/paraplegia) and severity (motor-complete/motor-incomplete) [19,20]. Tetraplegia was defined as lesions from level C2 to T1, whereas paraplegia was defined as lesions at level T2 or below. The AIS further categorizes SCI as complete or incomplete. Complete SCI was defined as the total absence of motor and sensory function, including sacral segments S4–5 (classified as AIS A). Conversely, incomplete SCI involves the preservation of motor or sensory function below the lesion site. Individuals with preserved sensory function but no motor function are categorized as AIS B. Individuals with preserved motor function below the lesion site, where more than half of the key muscles below the neurological level of injury score motor grades of 2 or lower, are designated as AIS C. Individuals with preserved motor function below the lesion site, with at least half of the key muscles below the neurological level of injury scoring motor grades of 3 or higher, are classified as AIS D. For this study, AIS A or B were considered “motor-complete”, whereas AIS C or D were deemed “motor-incomplete”.

Comorbidity was evaluated using the Charlson Comorbidity Index (CCI) [21]. Dysphagia was identified through a medical chart review. Individuals were classified as having dysphagia if they received tube feeding or texture-modified meals to support their swallowing function; otherwise, they were classified as not having dysphagia.

ADL was assessed using the Functional Independence Measure (FIM), Japanese version 3.0 [22,23]. The FIM consists of 18 tasks: 13 motor and 5 cognition tasks. Motor tasks include self-care tasks, such as eating, grooming, dressing, bathing, and toileting; excretory-related tasks, such as bladder and bowel management; transfers between bed, toilet, and bathtub; and mobility tasks, such as walking, wheelchair maneuvers, and using stairs. Cognitive tasks include communication tasks consisting of comprehension and expression and social cognitive tasks consisting of social interaction, problem-solving, and memory. Each task was scored on a seven-point scale (1–7) ranging from full assistance to independence. The total score for all thirteen items in the FIM motor task (FIM motor score) ranged from 13 to 91 points, the total score for all five items in the FIM cognition task (FIM cognition score) ranged from 5 to 35 points, and the total score for the FIM motor and cognition tasks (FIM total score) ranged from 18 to 126 points. Lower scores indicated less independence and greater dependence in ADL. Registered physical therapists, occupational therapists, nurses, and physicians jointly assessed the FIM based on the individual’s functional status on admission and monthly, during hospitalization. The FIM score assessed closest to the DXA scan date was used for the analysis.

A tape measure and electronic scale were used to assess body height and weight, respectively. This information was extracted from the medical charts. Body mass index (BMI) was determined by dividing body mass by height squared (kg/m^2^). Body composition measures, including the skeletal muscle mass index (SMI), lean tissue mass, percent body fat (%BF), fat tissue mass, and visceral adipose tissue (VAT) were assessed using whole-body DXA with Horizon A models (Hologic Inc., Marlborough, MA, USA) and analyzed using commercially available APEX software, version 5.6.04 (Hologic).

### 2.3. Outcome Measurements

The primary outcome measure was the FIM motor score. The secondary outcomes were the FIM cognition score, FIM total score, SMI, %BF, lean tissue mass, fat tissue mass, and VAT.

### 2.4. Criteria for Identifying Sarcopenia, Obesity, and Sarcopenic Obesity

Sarcopenia is diagnosed when low SMI, low hand grip strength, and/or low physical function (assessed by either the Short Physical Performance Battery [SPPB], six-meter walk, or five-time chair stand tests) are present [1]. However, these tests are often not feasible in individuals with SCI [24]. Hence, based on previous studies [13,14], only the SMI was used to identify sarcopenia. The SMI reference values were those recommended by the Asian Working Group for Sarcopenia 2019 consensus, with sarcopenia determined as <7.0 kg/m^2^ for men and <5.4 kg/m^2^ for women [1].

Obesity was determined based on criteria recommended by the American Association of Clinical Endocrinology [25,26]. Based on this criterion, obesity was identified when the %BF was greater than 25% in men and 35% in women.

There is no consensus regarding the diagnostic criteria for sarcopenic obesity. Based on previous studies, sarcopenic obesity was identified when the criteria for sarcopenia and obesity were met simultaneously [13,14].

### 2.5. Statistical Analysis

Categorical data are presented as numbers and percentages. Parametric data are reported as means and standard deviations, whereas nonparametric data are expressed as medians and interquartile ranges. Participants were divided into groups based on the presence of sarcopenic obesity. Normality and equality of variance were assessed using the Shapiro–Wilk and Levene’s tests, respectively. Depending on the variables, a *t*-test, Welch’s *t*-test, Mann–Whitney U-test, chi-square test, or Fisher’s exact test were used to compare the basic data and FIM scores between the groups.

In addition, a multiple regression analysis was performed to examine the association between SMI, %BF, and ADL. Age, sex (female = 0, male = 1), lesion level (tetraplegia = 0, paraplegia = 1), severity of injury (motor-complete = 0, motor-incomplete = 1), CCI, and duration of injury were considered as covariates. Multicollinearity was evaluated using the variance inflation factor (VIF), with VIF values ranging from 1 to 10 indicating no multicollinearity. Data were analyzed using IBM SPSS Statistics version 28.0 (IBM, Tokyo, Japan), except for the post hoc power analysis, which was performed using G*Power 3.1 [27,28]. The significance level was set at *p* < 0.05.

### 2.6. Ethical Considerations

This study adhered to the principles of the Declaration of Helsinki and the Ethical Guidelines for Medical and Health Research Involving Human Subjects. As this was a retrospective study, the opt-out method was employed, and the requirement of written informed consent from the participants was waived after notifying them of the study plan on the hospital website. The opt-out procedure allowed participants to cease their participation in this study at any time. The Ethics Committee of the Ibaraki Prefectural University of Health Sciences approved this study (approval number: e422; approval date: 20 December 2023).

## 3. Results

### 3.1. Participant Characteristics, Anthropometrics, and Body Composition Measures

The participants’ characteristics, anthropometrics, and body composition measures are depicted in Table 1. The median age was 63.5 [50.8–72.3] years, and 18.3% (*n* = 15) were female. The SCI was traumatic in 78.0% (*n* = 64) and non-traumatic in 22.0% (*n* = 18) of the participants. Causes of non-traumatic SCI included spinal cord infarction (*n* = 6), spinal epidural hematoma (*n* = 3), pyogenic spondylitis (*n* = 3), spinal epidural abscess (*n* = 1), transverse myelitis (*n* = 1), spinal cord hemorrhage (*n* = 1), and iatrogenesis of unknown cause (*n* = 3). The median duration of injury was 1698.5 [369.8–4123.5] days.

Among the participants, 76.80% (*n* = 63) had sarcopenia, 81.70% (*n* = 67) had obesity, and 62.2% (*n* = 51) had sarcopenic obesity. A comparison of participant characteristics and anthropometrics between the sarcopenic obesity and non-sarcopenic obesity groups revealed significant differences in sex, injury severity, and BMI. The proportion of female participants was significantly lower in the sarcopenic obesity group than in the non-sarcopenic obesity group (9.8% vs. 32.3%, *p* = 0.011). The proportion of motor-complete injuries was significantly higher in the sarcopenic obesity group than in the non-sarcopenic obesity group (60.8% vs. 25.8%, *p* = 0.002). The BMI of the sarcopenic obesity group was significantly lower than that of the non-sarcopenic obesity group (22.5 ± 2.8 vs. 25.3 ± 5.1 kg/m^2^, *p* = 0.007). 

The intergroup comparisons of body composition measures revealed that lean tissue mass-related indices were significantly lower in the sarcopenic obesity group than in the non-sarcopenic obesity group (lean tissue mass: 40.6 ± 6.6 vs. 44.6 ± 9.2 kg, *p* = 0.038; SMI: 5.4 ± 0.9 vs. 6.5 ± 1.2 kg/m^2^, *p* < 0.001). No significant differences were found in fat tissue mass-related indices between the two groups.

### 3.2. FIM Scores

The comparison of the FIM scores between the sarcopenic obesity and non-sarcopenic obesity groups is shown in Table 2. The FIM motor and total scores were significantly lower in the sarcopenic obesity group than those in the non-sarcopenic obesity group (motor scores: 54.0 [28.0–70.0] vs. 69.0 [47.0–80.0] points, *p* = 0.006; total score: 89.0 [62.0–105.0] vs. 104.0 [82.0–115.9] points, *p* = 0.007). The FIM cognition scores did not significantly differ between the two groups.

### 3.3. Association Between Body Composition Measures and ADL

The associations among SMI, %BF, and the FIM scores are shown in Table 3. Multicollinearity was not observed among the variables. SMI (β = 0.416, *p* < 0.001) and %BF (β = −0.325, *p* = 0.009) were independently associated with FIM motor scores. No significant associations were found between SMI, %BF, and the FIM cognition scores. SMI (β = 0.421, *p* < 0.001) and %BF (β = −0.313, *p* = 0.012) were independently associated with the FIM total scores.

### 3.4. Post Hoc Power Analysis

The results of the post hoc power analysis are presented in Table 4. In the multiple regression models with eight explanatory variables, the degree-of-freedom adjusted coefficients of determination (R^2^) ranged from 0.188 to 0.367 for Models 1 to 3. At a significance level of 0.05, an effect size (f^2^) of 0.232–0.580, and 82 cases, the power ranged from 85.9 to 100.0%.

## 4. Discussion

To the best of our knowledge, this is the first study to examine the relationship between DXA-measured lean and fat tissue mass-related indices and ADL in individuals with SCI. The findings revealed that SMI and %BF were independently associated with ADL regardless of age, sex, lesion injury, severity of injury, duration of injury, and comorbidities. The degree of association was mild, with a β of 0.416 for SMI and −0.325 for %BF, with SMI having a stronger effect. These results suggest that sarcopenic obesity, as identified by SMI and %BF, may be a risk factor for decreased ADL independence and increased ADL dependence in individuals with SCI.

### 4.1. Association Between SMI, %BF, and ADL

Sarcopenia negatively affects ADL. Studies have reported a negative association between sarcopenia and ADL in community-dwelling older individuals [18,29,30,31]. In addition, a study examining the association between sarcopenia and ADL in patients recovering from stroke, musculoskeletal diseases, and hospital-related deconditioning in a rehabilitation setting reported a negative association between sarcopenia and FIM motor scores [32]. Consistent with these reports, multiple regression analysis in our study revealed that the SMI was positively associated with the FIM motor scores. This result suggests that reduced SMI, comparable to sarcopenia, is a risk factor for decreased ADL independence in individuals with SCI.

Obesity may also have a negative impact on ADL. Previous studies identified obesity using BMI and reported a negative association between obesity and functional outcomes in individuals with SCI [33,34,35,36]. For example, Kao et al. investigated the relationship between BMI and ADL improvement in 3413 patients with traumatic SCI [36]. They reported that the improvement in FIM motor scores was poorer in the obese group than those in the underweight or normal-weight groups, and obesity was negatively associated with improvement in the FIM motor scores. Similarly, studies by Stenson et al. and Tian et al. reported a negative effect of obesity on ADL outcomes in patients with paraplegia on discharge from rehabilitation [34,35]. However, conflicting results have been reported in patients with tetraplegia [34,35,37]. Stenson et al. reported that the FIM self-care and mobility scores were not significantly different between obese and normal-weight patients with tetraplegia. Tian et al. also found no significant association between BMI categories and the FIM motor scores in patients with tetraplegia; the long-term FIM motor scores were higher in the overweight group than in the underweight or normal-weight groups [35]. Tanaka et al. reported a positive association between BMI and FIM efficiency in patients with cervical SCI admitted to a convalescent rehabilitation ward [37]. These results suggest that in individuals with tetraplegia, a higher BMI, being overweight, or being obese may have a positive effect on ADL.

Nevertheless, BMI may not accurately reflect the body composition of individuals with SCI because it cannot distinguish between skeletal muscle mass loss and fat tissue mass gain after SCI [10,13,38]. Hence, the relationship between obesity and ADL needs to be assessed based on the results of the body composition assessment. Kim et al. investigated the association between %BF and ADL in patients with degenerative lumbar spinal stenosis and reported a negative association between %BF and ADL [39]. Similarly, in the present study, the multiple regression analysis revealed a negative association between %BF and ADL independence. Specifically, increased %BF may inhibit ADL independence in individuals with SCI.

In addition to obesity, sarcopenic obesity may also inhibit ADL independence. Sarcopenic obesity has been reported to be negatively associated with ADL in community-dwelling older individuals [18,30]. Similarly, Matsushita et al. investigated this association in patients with stroke admitted to a rehabilitation ward and reported a negative association between sarcopenic obesity and the level of independence in ADL [5]. Furthermore, Yoshimura et al. investigated this association in patients with stroke, musculoskeletal diseases, and hospital-associated deconditioning in a post-acute rehabilitation setting, and reported a negative association between sarcopenic obesity, ADL improvement, and home discharge rates [4]. Consistent with these findings, this study found that the sarcopenic obesity group had significantly lower FIM motor and total scores than the non-sarcopenic obesity group. In addition, multiple regression analysis revealed that a lower SMI and higher %BF were significantly associated with lower FIM motor scores in individuals with SCI. In other words, sarcopenic obesity may impede ADL in individuals with SCI.

The link between sarcopenic obesity and ADL may be mediated by multiple mechanisms. First, a reduction in skeletal muscle mass is directly related to a decline in muscle function (strength), which can impair physical function [40,41]. Additionally, increased fat tissue mass resulting from reduced skeletal muscle mass, lower basal metabolism, decreased physical activity, and energy imbalance can negatively affect physical function [26]. This is because an increase in fat tissue mass is associated with insulin resistance, which is aggravated by the activation of inflammatory response pathways and the promotion of inflammatory cytokines such as tumor necrosis factor and interleukin-6, as well as hormones such as leptin, growth hormone, and insulin-like growth factor 1 [26]. Hyperinsulinemia associated with insulin resistance elevates serum myostatin levels [42]. As myostatin is a growth inhibitor in skeletal muscle, an increase in myostatin can accelerate skeletal muscle mass loss. Furthermore, intramuscular fat accumulation inhibits the regenerative capacity of skeletal muscles and promotes muscle fibrosis [26]. Changes in skeletal muscle structure may also cause the worsening of insulin resistance [26]. These intricate pathophysiological mechanisms may be closely related to the changes in body composition and diminished physical function observed after SCI.

Similarly, intricate mechanisms, including reduced physical activity, chronic inflammation, oxidative stress, and increased insulin resistance, are believed to contribute to cognitive decline, and an association between sarcopenic obesity and cognitive function decline has been reported in community-dwelling older individuals [43,44,45]. Additionally, Matsushita et al. recently reported this association in patients recovering from stroke in a convalescent rehabilitation ward [5]. However, we did not find this association in individuals with SCI, and no clear association was observed among the FIM cognition scores, SMI, and %BF. Patients with SCI and mild to severe cognitive impairment are often not considered for admission at our rehabilitation hospital, and participants with cognitive impairment were limited in our sample, which may have influenced this result. Hence, more studies are necessary to investigate the relationship between sarcopenic obesity and cognitive function in individuals with SCI.

### 4.2. Sarcopenic Obesity

Sarcopenic obesity is prevalent in individuals with chronic SCI. A study conducted with community-dwelling older people in Korea found that the prevalence of sarcopenia obesity defined using several criteria ranged from 1.3 to 20.3% in male individuals and 0.8 to 16.5% in female individuals [2]. By contrast, research by Pelletier et al. with community-dwelling Canadian individuals with chronic SCI revealed a much higher prevalence of 41.9% [13]. The current study showed an even higher rate of 62.2% among our participants. This difference might be attributed to the age disparity between the studies; Pelletier et al.‘s participants had a mean age of 33.5 years, whereas the median age in this study was 63.5 years. Aging is reported to be associated with reduced lean tissue mass and increased adiposity [46]. Other factors, such as differences in the criteria for defining sarcopenic obesity, race, ethnicity, and lifestyle, may have also influenced these results. Although direct comparisons are challenging due to these differences, individuals with SCI likely have a relatively high incidence of sarcopenic obesity.

Sarcopenic obesity is more prevalent in individuals with motor-complete injuries and in male individuals. Our study found that 60.8% of participants with sarcopenic obesity had motor-complete injuries, suggesting that this group may face an increased risk of developing sarcopenic obesity. This finding aligns with that of previous studies reporting higher %BF and lower lean indices in individuals with motor-complete injuries than in those with motor-incomplete injuries [13,47]. Additionally, sarcopenic obesity was more common in male participants. However, this study included a limited sample, particularly of female participants. Thus, further research with larger sample sizes is necessary to thoroughly investigate the risk factors associated with sarcopenic obesity and explore potential sex-based differences in its occurrence.

Finally, it is noteworthy that participants with sarcopenic obesity had a significantly lower BMI than those without this condition. The BMI was 22.5 kg/m^2^ and 25.3 kg/m^2^ in those with and without sarcopenic obesity, respectively. Nevertheless, %BF was comparably high between the two groups, with patients with sarcopenic obesity having 31.1% and non-sarcopenic individuals having 30.2%. This observation is consistent with those of previous studies, highlighting the limitations of using BMI as a tool to assess body composition in individuals with SCI [10,13,38].

### 4.3. Limitations and Future Prospects

This study has some limitations. First, it was a retrospective cross-sectional study conducted at a single institution, which may limit the generalizability of the results. Second, the number of participants was limited. Although the post hoc power analysis showed adequate power for multiple regression analysis, the number of explanatory variables used to estimate the partial regression coefficients was limited, and potential confounders may not have been considered. Third, a causal relationship between body composition and ADL could not be established using this study design. Hence, future prospective cohort studies should be planned with an increased number of participants and a larger number of potential confounding explanatory variables. Fourth, this study included legal adults aged ≥ 20 years, resulting in the inclusion of participants with a broad age spectrum. Although the effect of age was considered in the multiple regression analysis, the results may differ in samples with different age groups. Fifth, because there is no clear evidence as to whether sarcopenia in individuals with SCI can be assessed using the diagnostic criteria for the general population, this study did not assess skeletal muscle function (muscle strength or physical function). Only the SMI criterion was used to identify sarcopenia. For the general population, it is recommended to assess muscle strength (by handgrip strength testing) and physical function (by either the SPPB score, six-meter walk, or the five-time chair stand testing) [26,27]. However, these tests may not be feasible for individuals with SCI without potential statistical bias [28]. For example, muscle strength cannot be measured using a hand dynamometer in individuals with tetraplegia. In addition, handgrip strength may be higher in individuals with paraplegia than in normal participants because many paraplegic individuals depend on their upper limbs for ADL and use them more than normal participants do. Moreover, the SPPB, six-meter walk, and five-time chair stand tests cannot be performed by many individuals with SCI. Therefore, to accurately diagnose sarcopenia and sarcopenic obesity, alternative methods are needed to assess muscle function in individuals with SCI.

### 4.4. Clinical Implications

Despite these limitations, we found associations among decreased SMI, increased %BF, and reduced ADL independence in patients with chronic SCI. More than two-thirds had sarcopenic obesity, suggesting that these conditions are common among individuals with chronic SCI. The reduction in lean tissue mass and increase in %BF is reported to be associated with advancing age and longer injury duration [10,46]. As medical technology progresses and the population ages, the number of individuals with SCI may increase, potentially leading to a higher prevalence of sarcopenic obesity among individuals with SCI. This condition is not only connected to various health issues but also affects ADL, potentially negatively affecting the quality of life for those affected. Although neurogenic changes in body composition following SCI may be unavoidable, unfavorable alterations resulting from inactivity, immobility, and poor dietary habits are modifiable and should be managed. Early detection is necessary to prevent secondary complications associated with unfavorable changes in body composition. Routine body composition assessment is crucial for identifying those in need of preventive and therapeutic measures. Early detection and consideration of therapeutic interventions may help improve the physical function and health of individuals with SCI.

## 5. Conclusions

Decreased SMI and increased %BF were associated with reduced ADL independence in individuals with chronic SCI. Sarcopenic obesity was also prevalent in this group. Routine body composition assessments are warranted for early detection and consideration of therapeutic interventions to improve the physical function and health of individuals with SCI. More studies are necessary to investigate the relationship between body composition measures and physical function and to enhance the health of individuals with spinal cord injury.

## Figures and Tables

**Table 1:** Basic characteristics, anthropometrics, and body composition measures of the participants.

	Total (*n* = 82)		Sarcopenic Obesity (*n* = 51)		Non-Sarcopenic Obesity (*n* = 31)		*p*	Analysis	
Age (years)	63.5	(50.8–72.3)	64.0	(51.0–73.0)	61.0	(48.0–69.0)	0.494	b	
Sex, number of female participants	15	18.3%	5	9.8%	10	32.3%	0.011	a	*
Etiology, number with traumatic	64	78.0%	39	76.5%	25	80.6%	0.658	a	
Lesion level, number with tetraplegia	51	62.2%	30	58.8%	21	67.7%	0.419	a	
Severity of injury, number with complete injury	39	47.6%	31	60.8%	8	25.8%	0.002	a	*
Number with sarcopenia	63	76.8%	―	―	12	38.7%	―		
Number with obesity	67	81.7%	―	―	16	51.6%	―		
Duration of injury (days)	1698.5	(369.8–4123.5)	1729.0	(366.0–5113.0)	1668.0	(493.0–3636.0)	0.989	b	
CCI	2	(2.0–3.0)	2.0	(2.0–2.0)	2.0	(2.0–3.0)	0.195	b	
Anthropometrics									
Body height (cm)	167.3	(160.8–170.0)	166.8	±6.8	164.2	±9.4	0.154	c	
Body weight (kg)	62.8	(57.2–73.1)	62.7	±9.8	68.3	±15.1	0.072	d	
BMI (kg/m^2^)	22.8	(20.7–26.5)	22.5	±2.8	25.3	±5.1	0.007	d	*
Body composition									
Lean tissue mass (kg)	42.1	±7.9	40.6	±6.6	44.6	±9.2	0.038	d	*
SMI (kg/m^2^)	5.8	±1.1	5.4	±0.9	6.5	±1.2	<0.001	d	*
Fat tissue mass (kg)	19.2	(16.1–24.1)	19.0	(16.8–23.1)	21.2	(14.4–26.7)	0.966	b	
%BF (%)	31.8	±6.2	31.1	(28.2–35.4)	30.2	(23.7–37.8)	0.278	b	
VAT (cm^2^)	126.4	(85.4–160.8)	131.2	(92.2–157.4)	110.5	(77.5–167.2)	0.295	b	

Values are presented as *n* (%), mean ± SD, or median (IQR). Abbreviations: %BF, percent body fat; BMI, body mass index; CCI, Charlson Comorbidity Index; IQR, interquartile range; SMI, skeletal muscle mass index; SD, standard deviation; VAT, visceral adipose tissue. a, chi-square test; b, Mann–Whitney U test; c, *t*-test; d, Welch’s *t*-test; *n*, number of participants. * *p* < 0.05.

**Table 2:** Comparison of FIM scores between the sarcopenic obesity and non-sarcopenic obesity groups.

	Total (*n* = 82)		Sarcopenic Obesity (*n* = 51)		Non-Sarcopenic Obesity (*n* = 31)		*p*	
Motor score	60.5	(34.8–71.3)	54.0	(28.0–70.0)	69.0	(47.0–80.0)	0.006	*
Cognition score	35.0	(35.0–35.0)	35.0	(35.0–35.0)	35.0	(34.0–35.0)	0.707	
Total score	95.5	(69.8–106.3)	89.0	(62.0–105.0)	104.0	(82.0–115.0)	0.007	*

Values are presented as median (IQR). Abbreviations: IQR, interquartile range. *n*: number of participants. * *p* < 0.05.

**Table 3:** Association between body composition index (SMI and %BF) and the FIM scores.

	Model 1				Model 2				Model 3			
	Motor Score				Cognition Score				Total Score			
	β	*p*		VIF	β	*p*		VIF	β	*p*		VIF
(Constant)		0.010	*			<0.001	*			<0.001	*	
Sex (female/male)	−0.368	0.003	*	1.791	−0.218	0.109		1.791	−0.375	0.002	*	1.791
Age (years)	−0.132	0.177		1.193	−0.308	0.006	*	1.193	−0.153	0.117		1.193
Lesion level (tetraplegia/paraplegia)	0.281	0.006	*	1.265	0.102	0.367		1.265	0.280	0.006	*	1.265
Severity of injury (complete/incomplete)	0.179	0.137		1.813	−0.129	0.342		1.813	0.162	0.179		1.813
CCI	−0.061	0.523		1.138	−0.143	0.184		1.138	−0.071	0.456		1.138
Duration of injury (days)	0.072	0.458		1.181	0.247	0.026	*	1.181	0.090	0.350		1.181
SMI	0.416	<0.001	*	1.640	0.224	0.084		1.640	0.421	<0.001	*	1.640
%BF	−0.325	0.009	*	1.870	0.010	0.940		1.870	−0.313	0.012	*	1.870

β, standardized partial regression coefficient. Abbreviations: %BF, percent body fat; CCI, Charlson Comorbidity Index; SMI, skeletal muscle mass index; VIF, variance inflation factor. * *p* < 0.05.

**Table 4:** Post hoc power analysis of multiple regression models.

	Model 1	Model 2	Model 3
Adjusted R-square (R^2^)	0.367	0.188	0.365
Effect size (f^2^)	0.580	0.232	0.575
Power	100.0%	85.9%	99.9%

## Data Availability

Dataset is available on request from the authors.
